# On the Abuse of Abstemious Diet

**Published:** 1831-01-01

**Authors:** 


### ON THE ABUSE OF ABSTEMIOUS DIET. By Dr. CAFORT.

[From the Mem. des Hôpitaux du Midi.]

IF great mischief is daily done to the chylopoietic viscera by gourmandising and ingurgitation, so there is no doubt that some injury accrues from running to the opposite extreme of starvation. Not long ago we presented our readers with an interesting memoir of M. Barras —gastralgia mistaken for gastritis— and we believe that since the theory of Broussais came in vogue on the Continent, great and sometimes irreparable mischief is done by attributing every painful affection of the stomach to inflammation of its coats. The author of the present paper appears to have been one of the sufferers from the gastro-mania of the day, and it may not be uninstructive to glance at his own case.

Being in Paris in the year 1824, he became affected with "chronic gastritis," which, not being attended to, made progress till the succeeding year. The symptoms under which Dr. Cafort laboured were as follow:—"loss of appetite—pain in the epigastrium augmented by walking, especially if the foot struck against a stone—mouth pasty in the mornings—tongue red at the sides and white in the middle—gums spongy and hæmorrhagic—aphthæ—constipation—fæcal matters in hard balls—great lassitude after food—disinclination to exercise, &c." Towards the close of the year these symptoms were aggravated by some moral afflictions and by great fatigue which he underwent in literary avocations to keep his thoughts from dwelling on his personal grievances. At length being greatly harrassed by the gastric affection, he began to attend to treatment. He first applied leeches to the epigastrium, which gave relief, but did not cure him. He then put himself on a most rigid regimen—namely a small quantity of milk daily, without any other food. After a time he added to the milk some grated potatoe. Under this regimen, continued for two months, the epigastric pains entirely ceased; but the appetite not returning, he dared not to increase his food, fearing a return of the GASTRITIS. He therefore persevered, at the expense of a diminution of strength, emaciation, deadly pallor, and great want of sleep. To these were added distressing palpitations of the heart on awaking from sleep, followed by tendency to syncope. In the course of time, the pulse became intermittent, and the palpitations were daily as well as nightly distressing. He happened to perceive that the intermissions of the pulse, the palpitations, and the tendency to syncope occurred most when the digestion of food was entirely over, and the stomach completely empty. This discovery set him thinking and reasoning on the phenomena of starvation, and he soon was able to explain all the symptoms under which he laboured after the cessation of the gastric pains, upon the principle of defective nutrition. He now began to gradually and progressively increase the scale of his diet, and to vary the quality thereof—and from that moment all the distressing symptoms began to retreat, and the Doctor, during a long tour which he then made, recovered from a disease which he fully expected would conduct him to the tomb.

Now, in the first place, we much doubt whether the disease was GASTRITIS at all. We are quite sure, however, that there was a considerable portion of gastralgia combined with it. In the second place, Dr. Cafort fell into an error in putting himself upon a small quantity of milk for his sole nutriment —and thirdly he fell into a still greater error when he pursued the same system of starvation after the gastric pains had ceased, and consequently after the principal object of the abstemious diet had been obtained. Had he put himself upon farinaceous food with beef tea at the beginning, and gradually increased the scale of sustenance, and the quality of the food, after the epigastric uneasiness had given way, he would have been cured in a short time by diet alone. Had very gentle aperients combined or not with the mildest bitters, and assisted by efficient counter-irritation, been employed, the recovery would have been still more rapid. The case shews the errors into which the doctrines of Broussais lead our brethren on the Continent. In our own country, indeed, the doctrines of a celebrated gastro-hepatic teacher and practitioner have done still more mischief than the starving system of Broussais! The eternal calomel at night and black draught in the morning, have played their part very effectually, and may well triumph over the Broussaian visions which are more frequently harmless than injurious.

